# Protecting FPGA-Based Cryptohardware Implementations from Fault Attacks Using ADCs

**DOI:** 10.3390/s24051598

**Published:** 2024-02-29

**Authors:** Francisco Eugenio Potestad-Ordóñez, Alejandro Casado-Galán, Erica Tena-Sánchez

**Affiliations:** 1 Escuela Politécnica Superior, Universidad de Sevilla, 41011 Seville, Spain; etena@us.es; 2 Instituto de Microelectrónica de Sevilla, IMSE-CNM (CSIC; Universidad de Sevilla), 41092 Seville, Spain; casado@imse-cnm.csic.es

**Keywords:** hardware security, voltage attack, temperature attack, electromagnetic attack, countermeasures, FPGA

## Abstract

The majority of data exchanged between connected devices are confidential and must be protected against unauthorized access. To ensure data protection, so-called cryptographic algorithms are used. These algorithms have proven to be mathematically secure against brute force due to the key length, but their physical implementations are vulnerable against physical attacks. The physical implementation of these algorithms can result in the disclosure of information that can be used to access confidential data. Some of the most powerful hardware attacks presented in the literature are called fault injection attacks. These attacks involve introducing a malfunction into the normal operation of the device and then analyzing the data obtained by comparing them with the expected behavior. Some of the most common methods for injecting faults are the variation of the supply voltage and temperature or the injection of electromagnetic pulses. In this paper, a hardware design methodology using analog-to-digital converters (ADCs) is presented to detect attacks on cryptocircuits and prevent information leakage during fault injection attacks. To assess the effectiveness of the proposed design approach, FPGA-based ADC modules were designed that detect changes in temperature and supply voltage. Two setups were implemented to test the scheme against voltage and temperature variations and injections of electromagnetic pulses. The results obtained demonstrate that, in 100% of the cases, when the correct operating voltage and temperature range were established, the detectors could activate an alarm signal when the cryptographic module was attacked, thus avoiding confidential information leakage and protecting data from being exploited.

## 1. Introduction

The proliferation of the Internet of Things (IoT) has caused a rapid and significant increase in the number of interconnected devices. These interconnected devices process a large amount of data, most of which are sensitive data from users. Due to the methods that hackers are constantly developing to gain access to users’ secret information, protecting sensitive data and countering attacks by third parties has become a constant challenge. Recent research has highlighted the importance of analyzing the security of IoT solutions in their various design stages [1].

Mahmoud et al. [2] and Xu et al. [3] pointed out the importance of and challenges related to security in the IoT, highlighting secure implementations under strong constraints of power consumption and area, among other considerations. The creation of new cryptographic algorithms that can safeguard data and meet the stringent requirements of applications is an ongoing process. One of the solutions proposed in [4] was the use of so-called lightweight cryptography as a solution to security problems and the use of solutions whose cost has the lowest possible impact on implementation. Finally, Ref. [5] warned of the potential dangers of not considering the security of embedded applications used in this field.

A cryptographic module is deemed secure in practice if there is no known attack that can successfully break it in a reasonable amount of time and with a reasonable amount of computing power. Although algorithms are mathematically secure, their physical implementation on hardware can be exploited by third parties to reveal confidential information through side-channel attacks (SCAs) or fault injection attacks (FIAs) [6,7].

**Figure 1:**
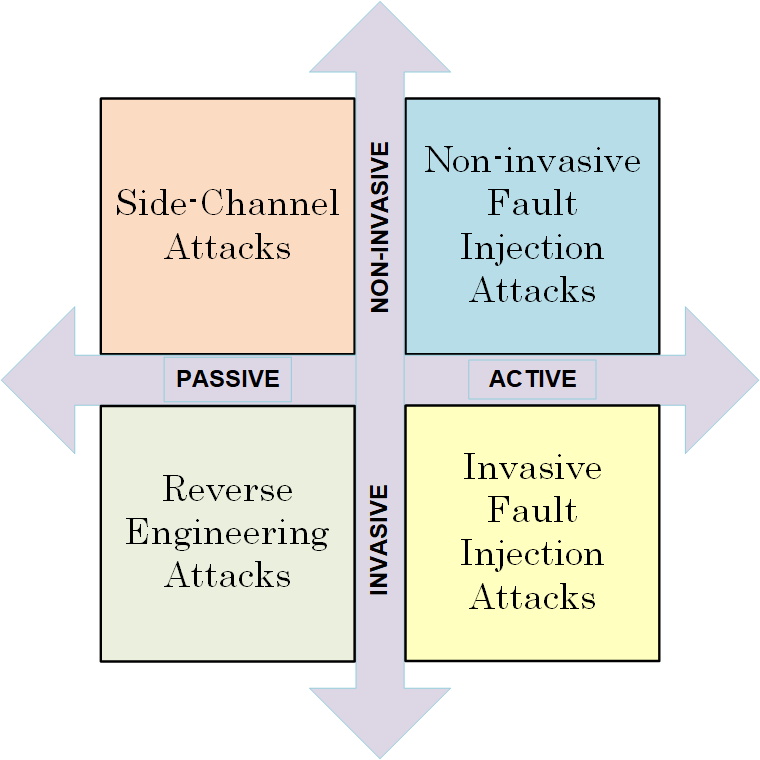
Attack classification.

There are various attack techniques that vary greatly in terms of the execution cost and time, equipment needed, and expertise required. These two characteristics can be used to classify them as active or passive and invasive or non-invasive [7,8]. Figure 1 summarizes the attack classification. Invasive attacks involve manipulating the device, typically by unpacking the chip and gaining access to its internal layers. Non-invasive attacks, on the other hand, collect data from the device (such as accessible I/O, power consumption, and execution time) without making any changes. In a passive attack, confidential data are exposed while the cryptographic system is functioning correctly by examining side-channel information such as power usage, timing, or electromagnetic radiation. However, an active attack changes the functionality of the device during operation, manipulating its input, power supply, or environmental conditions such as temperature. The malfunctioning of the process and the results it produces can be used to uncover the secret key. Invasive attacks, whether passive or active, are costly in terms of time and money and can often cause irreparable damage to the cryptographic device. Non-invasive attacks are a major concern for the cryptographic community due to their low cost, minimal equipment requirements, and high success rate.

With this in mind, great effort has been put into implementing cryptographic algorithms securely and efficiently on hardware platforms. It is important to point out that this paper concentrates on private-key ciphers, yet there are numerous studies that have concentrated on the implementation of public-key algorithms and their ability to withstand attacks from third parties. As evidence of this, there have been studies on effective executions of public-key algorithms such as the elliptic curve and RSA [9,10,11,12], as well as theoretical attacks [13,14,15]. Consequently, this study concentrates on the implementation of a security system on private-key encryption systems, while public-key encryption systems are not included. In this paper, we focus on non-invasive active attacks, specifically those that aim to manipulate the supply voltages and the operating temperature of the circuit that implements a cryptographic algorithm, and electromagnetic attacks. The proposed methodology enables one to implement a system that can identify these kinds of attacks and prevent the potential retrieval of confidential data stored in the circuit. The Advanced Encryption Standard (AES) is employed as a test vehicle. This countermeasure is not specifically designed for the AES encryption algorithm; it is used just as an example. It is a system that works in parallel and can be applied to any other cryptographic circuit or hardware-based cryptographic element, since it detects attacks on the hardware rather than the algorithm that is implemented in it. Therefore, this scheme can be applied in parallel to protect any other cipher implemented on the hardware.

The main objective of this work is the presentation and evaluation of a detection scheme methodology based on analog-to-digital converters to protect cryptohardware implementations. The contributions of the paper are the following:

- The classification of the most relevant types of attacks on physical implementations, focusing on attacks by the manipulation of supply voltage and temperature and the injection of electromagnetic pulses.
- The proposal of a methodology for the design of the detection scheme based on analog-to-digital converters, serving as a countermeasure to prevent information leakage.
- The development of two experimental case studies to evaluate the proposed design methodology. One focused on voltage and temperature manipulation. The other pertained to the injection of electromagnetic pulses.
- The comparison of the results obtained from the proposed system with the schemes reported in the literature.

The remainder of the paper is organized as follows. Section 2 presents an overview of the AES used as a test vehicle and the physical implementation attacks reported in the literature, focusing on voltage, temperature, and electromagnetic (EM) radiation. Section 3 describes the proposed design methodology to detect attacks based on temperature, voltage, and EM radiation. Section 4 presents the setups used to test the design methodology, the results obtained, and a comparison with previous schemes reported in the literature. Finally, Section 5 presents the conclusions derived from this work.

## 2. Physical Fault Injection Implementation Attacks

In the case of non-invasive active attacks based on fault injection, the attacker maliciously aims to cause transitory faults in the operations of cryptographic algorithms. Faults must not be permanent, as this would render the circuit unusable and eliminate the possibility of a differential study. With this in mind, the attacker will try to determine the erroneous behavior under different types of fault, which depends on the algorithm under attack, and compare it with the correct behavior of the circuit. This mathematical comparison is known as Differential Fault Analysis (DFA) [16] and allows one to establish the relationship between the produced faults and the internal information of the cipher. Depending on the encryption algorithm, the desired faults will be of different types: single-bit, multi-bit, single-byte, multi-byte, faults in the same byte, faults in different bytes, or even random faults. In the case of the AES cipher, analyses can be found in the following references [17,18,19,20]. These differ as to the place where the fault must be inserted, such as the state matrix, S-box operation, and KeySchedule. In addition, the differential analysis will differ depending on the encryption or decryption round in which the fault is inserted. Table 1 shows the classification of DFA attacks on the AES, considering if the attack is performed on the state, the S-box, or KeySchedule; the type of fault; and the round where the fault must be injected.

In short, the AES encryption scheme [21] is the official standard set by the National Institute of Standards and Technology (NIST) to replace the Data Encryption Standard (DES). It employs the Rijndael algorithm with input blocks of 128 bits. The AES conducts the input transformation across several rounds, with the number of rounds varying based on the key size selected (10 rounds for 128 bits, 12 rounds for 192 bits, and 14 rounds for 256 bits). The rounds involve manipulating the input state *S*, which is 16 bytes in size, using the SubBytes(), ShiftRows(), MixColumn(), and AddRoundKey() operations. In our experiments, we focused on the 128-bit key setup. This cipher is the most widely used and implemented in the vast majority of technologies because it is the most widely used standard for the symmetric block ciphering of information.

Regarding attacks on the AES cipher, the two main works are those presented by Giraud [17] and Dussart [18]. The first one established the assumptions that an attacker must follow in order to recover the secret key contained in the cryptocircuit when a single bit-level fault is injected into the state matrix. These attacks are carried out on the eighth round of encryption. On the other hand, the second paper presented a differential analysis based on the injection of multi-bit faults in the same byte of the state matrix. In this case, the attacks are performed from the seventh round and before the ninth round, prior to the MixColumn() operation. These were the first DFA attacks on the AES, but after them many more appeared continuously, modifying the assumptions of the attacks and the type, round, and location of the fault necessary to compromise the security of the cipher [19,20].

### 2.1. Voltage and Temperature Attacks

**Figure 2:**
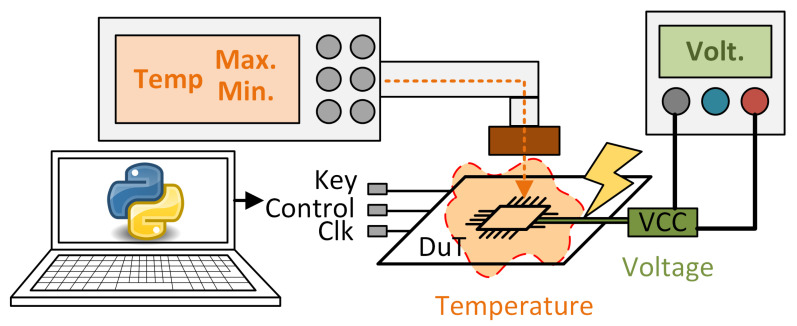
Voltage and temperature attack representation.

In the case of the supply voltage, an increase or decrease in the voltage supply to the chip above the tolerance level of the devices (typically 10%) can cause faults in the combinational operations or in the bits stored in the flip-flops (FFs). These faults can affect part of the circuit or cause widespread faults [22]. Additionally, tampering with the power supply can lead more easily to fault injection. In [23], the authors showed how by manipulating the supply voltage, fault injection through clock manipulation is made simpler, without the need to drive the circuit to extremes ar which it is more difficult for the attacker to achieve effective faults in cryptocircuits. A representative scheme of an attack modifying the circuit supply voltage can be seen in Figure 2.

When considering temperature attacks, exceeding the temperature range specified by chip manufacturers for proper operation can deliberately induce faults in the chip. By configuring the chip temperature to a level at which write operations are functional while reads are not, or vice versa, multiple attacks can be launched. Various kinds of faults can occur based on the susceptibility of the components to temperature. Typically, the attacker lacks specific control over the exact type of fault that will occur [24,25]. An illustrative example of an attack involving the manipulation of the circuit’s ambient temperature is depicted in Figure 2.

### 2.2. Electromagnetic Attacks

**Figure 3:**
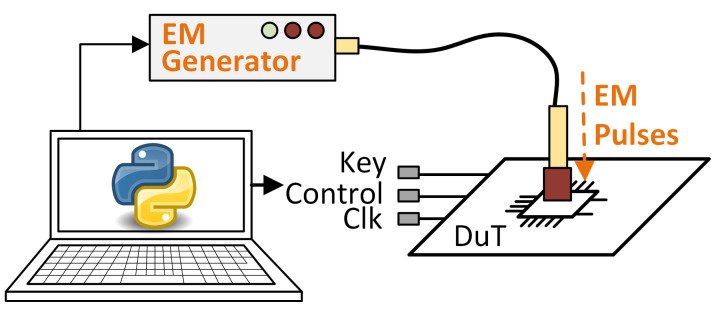
Electromagnetic attack representation.

Electromagnetic Fault Injection (EMFI) attacks are based on the introduction of errors in an integrated circuit using an electromagnetic pulse (EMP). When the electromagnetic field of the EMP penetrates the device, it produces anomalous voltage differences and currents within the components of the circuit. A representative scheme of an attack using an electromagnetic pulse injector can be seen in Figure 3. Inducing Foucault currents on the chip surface can cause a fault of up to a single bit [26]. In addition to the security aspect, there is also the characterization and study of the resilience of integrated circuits to fault insertions produced by the environment itself, such as electromagnetic interference or cosmic rays, when circuits are used in space applications [27]. However, the use of passive protections, such as passive shields provided by metal layers that cover sensitive parts of the chip, makes the injection of EM faults more difficult because a very high level of precision is required [6].

## 3. Design Methodology to Protect against Attacks

Taking into account the attacks mentioned above, the proposed solution uses the Xilinx Analog-to-Digital Converters (XADCs) provided by Xilinx in its Field-Programmable Gate Array (FPGA) devices. The XADC is a basic building block that enables analog mixed-signal (AMS) functionality. By combining analog blocks with programmable logic, it is possible to create customized analog interfaces for a wide range of applications.

The possible applications that can be developed with these components include the reading and monitoring of the analog values of the operating voltages of the device. Therefore, in this work, this component was used to establish operating ranges outside of which the system will understand that the device is being maliciously manipulated and will therefore activate an error signal that will allow the system to detect that it is being attacked.

**Figure 4:**
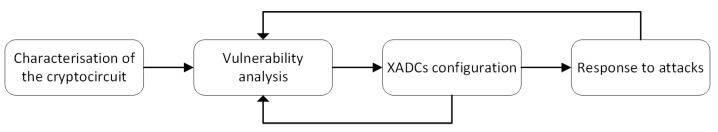
Design methodology process.

With the aim of designing a secure protection against these types of attacks, it is necessary to follow a design methodology that allows for the most efficient scheme. A schematic representation of the design methodology is presented in Figure 4. Firstly, it is necessary to characterize the cryptocircuit that is to be protected. Therefore, the maximum frequency, voltage supply, and temperature characteristics must be established, following the manufacturer’s data sheets. Once these parameters have been established, it is necessary to determine the possible vulnerabilities of the physical implementation, namely, the layout, power supply lines, etc. Thirdly, the designer must configure the XADC to cover the voltage and temperature ranges that are considered correct. At this point, out-of-range values are the aim of attackers when a fault is desired. Finally, a response must be selected against the manipulation of the circuit. This response could be different depending on the attack scenario considered. For example, responses to Ineffective Fault Attacks (IFAs) will be different from the responses to DFA attacks.

**Figure 5:**
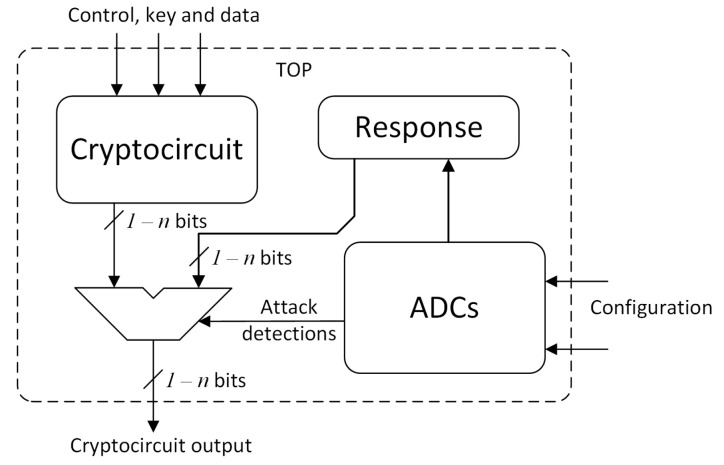
Schematic representation of the top module of protection.

In response to the attack, a scheme is proposed, as shown in Figure 5. Note that the cryptocircuit identifier is used since the system can be implemented to protect different cryptocircuits. In this scheme, the encryption algorithm and the XADC, which monitors the voltages and temperatures within the set range, are working in parallel. As long as the protection does not detect any anomalous state, the cryptocircuit can operate normally and output the encrypted data without any interference. However, if the protection system detects that the system is being attacked by any of the techniques described above, it generates an alarm signal that allows for the activation of the response to the attack and alerts the system or user of this cipher so that they are aware of the situation. This alarm would allow a root of trust that works independently, encrypting the information, to know the scenario in which it finds itself and that its security is compromised.

When the system detects that it is under attack, the alarm signal is triggered, and the response is activated. In this scheme, the response consists of writing a zero value in the output. With this response, an attacker cannot know whether the attack was effective or not due to the fact that any data related to the data stored or processed during encryption or decryption are given in the output, completely blocking DFA or IFA attacks.

It should be noted that FPGA ADCs have both internal and external inputs. In our case, we used internal signals that monitor the voltage and temperature ranges of the device. These inputs are not accessible from the outside, and, therefore, if an attacker tries to manipulate them, in addition to the enormous complexity that this would entail, any manipulation would cause the device to be out of its operating ranges, and thus the alarms would be triggered. Note that the inputs shown in Figure 5 denoted as *Configuration* are only accessible during the design process.

## 4. Setups and Results

**Figure 6:**
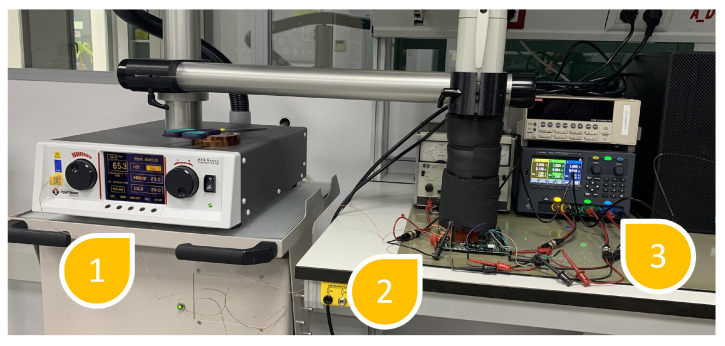
Experimental setup for testing voltage and temperature schemes.

**Figure 7:**
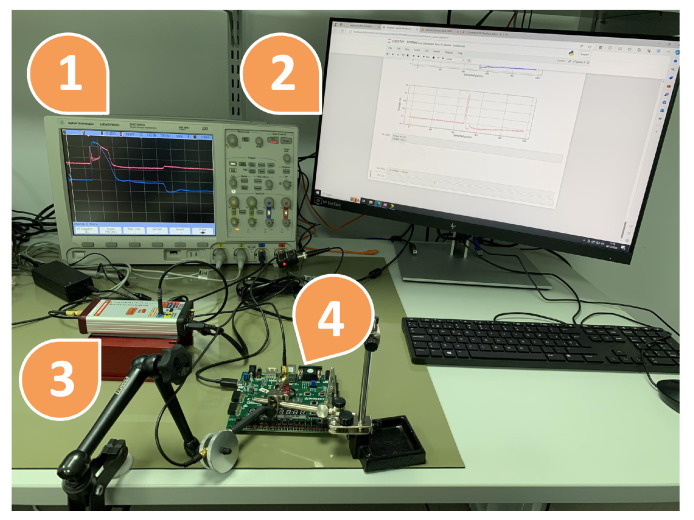
Experimental setup for testing electromagnetic scheme: (1) oscilloscope, (2) PC, (3) ChipSHOUTER, (4) Nexys A7 board.

To test the performance of this protection, a Xilinx Nexys 4 board with an Artix 7 100T FPGA (Xilinx Inc. from San Jose, CA, USA) was used. In addition to the board, different tools were used: a Thermonics ATS-505-S-2 (Temptronic Corporation, Mansfield, MA, USA) temperature control system, a Keysight e36312A (Keysight Technologies, Santa Rosa, CA, USA) power supply, an Agilent InfiniiVision DSO7054A oscilloscope (Agilent, Santa Clara, CA, USA) with 4 G/samples and a bandwidth of 500 MHz, and a NewAE EM pulse generator called ChipSHOUTER (NewAE Technology Inc., Dartmouth, Canada). Both setups can be observed in Figure 6 and Figure 7.

Different devices can be used to induce an EMP. In this work, we used the ChipSHOUTER (CW520) from NewAE Technology. This device charges a bank of capacitors that, when fired, are discharged through a probe tip, producing a short-duration high-intensity EMP in the vicinity of the tip. These probe tips are made of a conductor coil with a ferrite core inside that creates a (mainly) magnetic field. The voltage at which the capacitors can be charged and the approximate duration of the EMP are parameters that can be adjusted via a digital interface with a PC. The voltage is directly proportional to the intensity of the magnetic field produced, and the duration of the pulse also corresponds to the duration of the electromagnetic perturbation. In order to characterize the EMP, we placed a probe from the Rohde&Schwarz HZ-15 near-field electromagnetic probes kit (Rohde & Schwar, Munich, Germany) near the coil of the ChipSHOUTER. This probe measured the magnetic field inside its own coil, and the variation over time was recorded with an oscilloscope.

**Figure 8:**
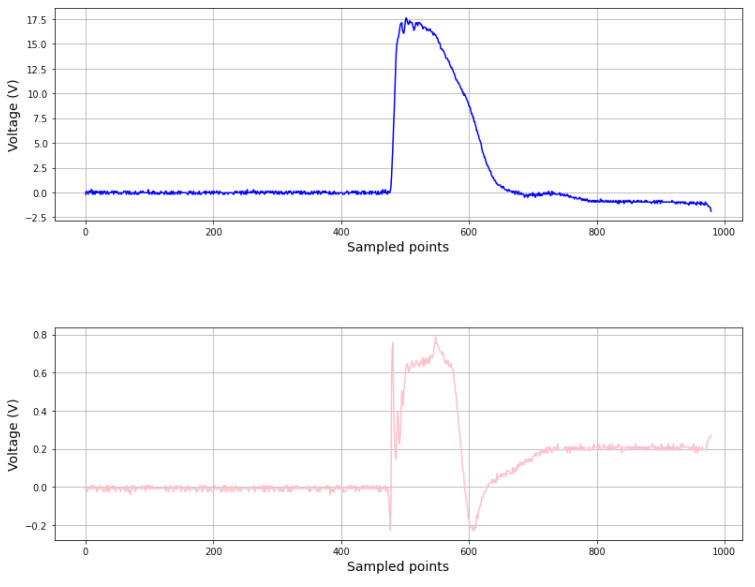
Comparison between the voltage levels of the ChipSHOUTER (**top**) and the magnetic field measured at the probe tip (**bottom**).

In Figure 8, we can see how the magnetic field induced near the probe tip corresponds to the variation in the voltage inside the ChipSHOUTER. We can see that the aspects of both traces are similar. The main differences are at the start and the end of the pulse generation. At first, since the voltage scales very fast, we can see a spike in the magnetic field produced by the quick variation. Then, the magnetic field is more or less constant during the pulse and relaxes to another constant value slightly different from zero. This could be because a current remained in the coil of the probe tip, and it produced a less intense magnetic field until it disappeared. This final relaxation to the initial state was much longer than the pulse, but it barely produced an alteration in the attacked signals.

We can infer then that the current and voltage variation induced by the EMP were higher the faster the intensity of the magnetic field changed. This makes sense if we observe Faraday’s law of induction (Equation (1)), which states that the electromotive force produced along a closed path is proportional to the rate of change of the magnetic flux enclosed by the path:

(1) $$\varepsilon =-\frac{d\varphi_{B}}{dt},$$

where ε is the electromotive force, and $\varphi_{B}$ is the magnetic flux.

### 4.1. Voltage and Temperature Setup

Figure 6 presents the configuration for the manipulation of temperature and voltage ((1) Thermonics, (2) Nexys board, and (3) supply voltage). In the case of the power supply voltage, the main power supply voltage, $V_{CCINT}$; the memory voltages of Random-Access Memory (RAM), $V_{CCBRAM}$; and the internal auxiliary power supply voltage of the FPGA itself, $V_{CCAUX}$, are protected against malicious variations. The values established as performance limits were those established following the manufacturer’s characterization tests. Specifically, for fault detection, the established ranges within which operation is considered correct are those presented in Table 2. Outside of these ranges, the protection scheme considers the device to be under attack.

**Figure 9:**
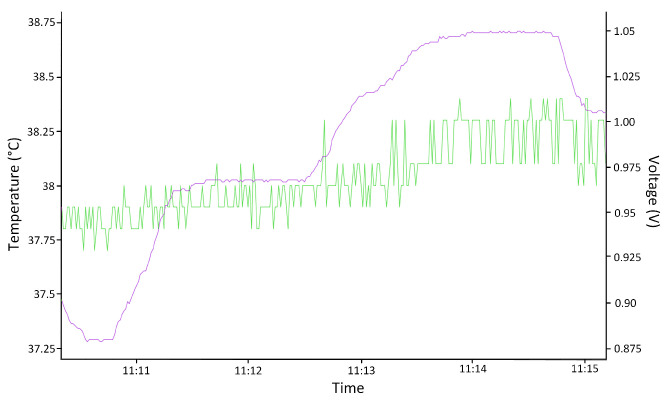
Screenshot of the voltage test outside the established ranges. Purple: Voltage; Green: Temperature.

For the voltage case, it is necessary to consider tools (2) and (3) in Figure 6. Figure 9 shows the result of the monitoring of voltage variation using the Xilinx Vivado (Xilinx Inc. from San Jose, CA, USA) tool. The supply voltage was modified to obtain a voltage outside the ranges (purple line), with a minimum of 0.87 V and a maximum of 1.06 V. Notice that the green line is the temperature variation of the circuit under these conditions.

For the case of temperature variations, the same scheme was used as for voltage variations. In this case, the XADC was configured to obtain the operating temperature values of the device and to check if the device was outside the normal operating ranges. For this purpose, a temperature range was defined for which the device should operate correctly and outside of which the system is considered to be under attack. The temperature range considered as normal operation is between 60 °C and 0 °C. This range was determined by the operating ranges given by the manufacturer, considering that our experimental application’s maximum temperature was fixed at 60 °C for the protection of the laboratory equipment and the device under test. This range was established according to the characterization data, which were considered normal behavior for a circuit in a typical work process.

### 4.2. Voltage and Temperature Results

For this test, the Xilinx Nexys 4 board, Figure 6 (2); a Thermonics ATS-505-S-2 temperature control system, Figure 6 (1); and a computer with Xilinx Vivado software were used to monitor the results.

**Figure 10:**
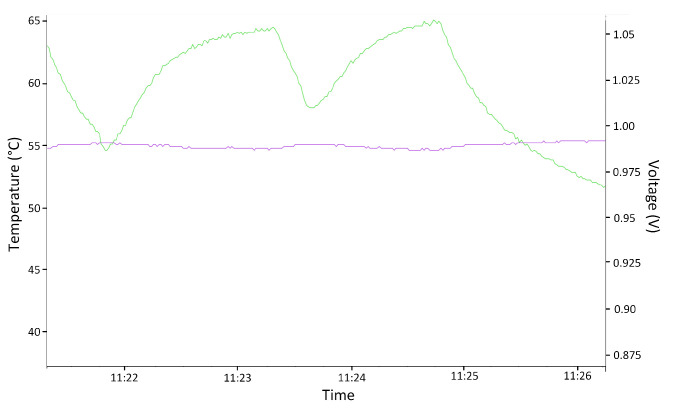
Screenshot of the temperature test outside the established range. Purple: Voltage; Green: Temperature.

In this case, only the temperature above the normal operating range could be tested, since subjecting the system to temperatures that were too low could produce small frozen water spots and irreversibly damage the device. Figure 10 shows the result of monitoring the temperature variation using the Xilinx Vivado tool. The temperature was modified to obtain a temperature outside the range (green line), which was the maximum value at 65.5 °C. The test again corroborated that the error signal was correctly triggered by turning on an LED on the board when the temperature was out of range. Note that the purple line is the voltage supply of the circuit in this test.

### 4.3. Electromagnetic Setup

**Figure 11:**
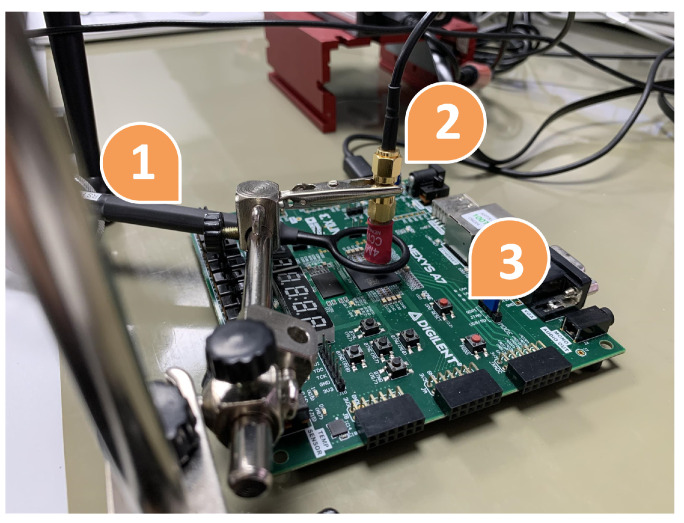
Details of experimental setup for testing electromagnetic scheme: (1) near-field magnetic probe, (2) probe tip of the ChipSHOUTER directly pointing at the Artix-7 FPGA, (3) Nexys A7 board.

In Figure 7, the EM fault attack setup can be observed. Figure 11 presents in more detail the same setup for EM attacks. In Figure 7, (1) is the Agilent InfiniiVision DSO7054A oscilloscope, (2) is a personal computer, (3) is the ChipSHOUTER, and (4) is the Nexys board. In Figure 11, (1) is a Rohde&Schwarz HZ-15, (2) is the probe tip, and (3) is the Nexys board. The main objective was to test whether inserting an EMP into the FPGA would detect a voltage rise (or fall) that could be detected by the XADC. For this, a 4 mm ChipSHOUTER probe tip (diameter of the ferrite core) was used directly above the FPGA encapsulation (approximately 1 mm). The magnetic field probe surrounding the probe tip of the ChipSHOUTER was used to measure the injected magnetic field. More specifically, this probe measured the component of the magnetic field vector that was perpendicular to the plane where the probe coil was located.

### 4.4. Electromagnetic Results

The success of an attack depended on the intensity of the pulse and the position along the XY plane of the FPGA of the ChipSHOUTER probe tip. There were some areas where only one pulse was needed to detect an anomalous voltage change in the FPGA by the XADC, while in other areas the injection of an EMP would reset and clear the whole FPGA.

**Figure 12:**
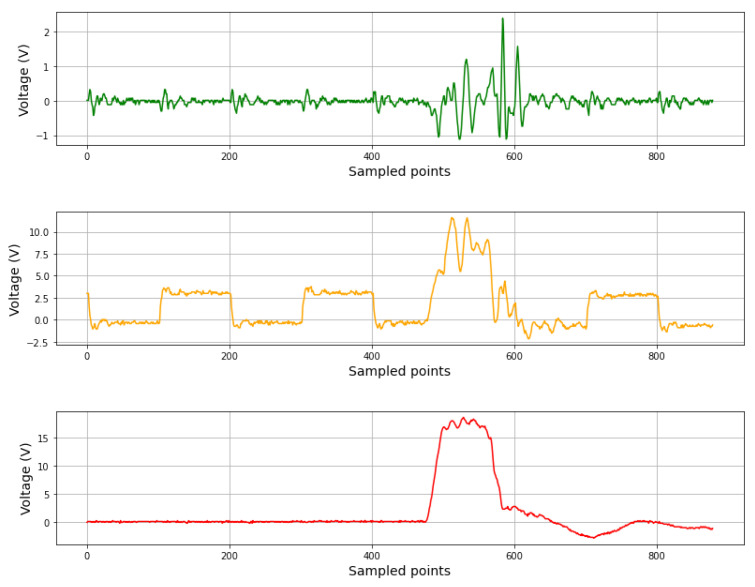
Result of an EM attack over the FPGA. Signal with zero logic value (green), FPGA clock signal (orange), and EM pulse injection (red).

The aim of the scheme was to determine whether pulse injection affected the internal voltage values of the FPGA. As can be seen in Figure 12, the injection of EM pulses into a zero logic signal and a clock signal altered the values of these signals, producing a disturbance of more than two volts. This test was performed over the external signals because the internal FPGA signal could not be sampled by the oscilloscope. These disturbances could be sampled by the XADC as variations of the voltage and, therefore, could detect EM attacks. To ascertain whether the EMP produced a voltage change outside of the typical values, circuitry was placed at the ADC output to sample its value each clock cycle (the clock frequency was 100 MHz). If a voltage rise or fall was detected, then the alarm was triggered. As a result, pulse injection was detected in the tests, allowing us to determine that the EM pulses altered the internal voltage and therefore were detectable by the proposed scheme. In these cases, the output of the criptocircuit was zero. The efficiency of the proposed protection scheme was 100% (all effective attacks were detected) in those cases where the injected fault caused no clearing or resetting of the FPGA.

As can be seen, the use of this methodology allows one to determine whether the system is being attacked by any of the three attack methods, representing a single detection and response scheme necessary to neutralize these attacks. As soon as the converter determines that the system is under attack, the circuit will not leak information, since it will not respond. Hence, the attacker cannot have access to the erroneous operation produced by the different fault injections into the cryptographic algorithm, and thus the attacker is unable to perform the differential analysis.

### 4.5. Comparison with Other Protection Schemes

With the aim of properly analyzing the methodology and protection proposed, Table 3 presents a summary of some schemes proposed in the literature for the cryptocircuit AES. As can be seen, there are different types of protections, such as information redundancy, hardware redundancy, temporal redundancy, and combined approaches. It should be noted that our protection does not belong to any of the above groups but can be compared with them in order to achieve an overview of their penalties. Table 3 presents the area overhead, frequency degradation, attack detection, and technology with which the schemes were implemented.

It is possible to establish a hierarchy of attack detection based on the fault coverage of the reported schemes, which can be compared with our scheme. On the other hand, if references [28,29,30,31,32,33,34] are considered, the table shows that these schemes are based on information redundancy, i.e., the protections add additional information to the data processed by the encryption circuit to detect if there has been any change. From this group, it is possible to see that the impact in terms of area is very high in the schemes of [29,30,31,33], which have at least 40% extra area. However, if the scheme whose area penalty is lower [28] is considered, it can be seen that its frequency degradation is very high, reaching 30%. In [35], a protection based on hardware redundancy was presented. This scheme adds part of the operations redundantly and compares the results to look for any error injection. Despite obtaining the same result for frequency degradation as the scheme presented in this paper, its area degradation is very high compared to the scheme proposed in this work, reaching an additional 86%. On the other hand, the scheme based on temporal redundancy in [36] built its protection on rerunning certain operations in search of discrepancies. As can be seen, its area degradation is slightly higher than our proposal, but, on the other hand, its frequency penalty is much higher, reaching 17%. Finally, the schemes in [37,38] employ a combination of protections. It can be observed that both have a high cost in area and their results in terms of frequency degradation are higher than the presented scheme, reaching up to 22%. On the other hand, if attack detection is taken into account, only in the cases of [35,36,37] and the proposed scheme was 100% reached. Among these, the scheme of [36] was the closest in terms of area consumption, with an additional 6%. However, as mentioned above, its frequency penalty is much higher.

Finally, it should be noted that the proposed solution could be applied in parallel with any of the other protection schemes, as it operates completely independently of the circuits and any other protection countermeasures. Furthermore, the use of this system makes it possible to determine not only whether the system is under attack but also whether an extreme situation is occurring that could cause the circuit to malfunction.

## 5. Conclusions

Due to the constant development and sophistication of attacks on hardware implementations of cryptographic elements, it is necessary to develop systems to protect user information from malicious third parties. To minimize these attacks, countermeasures or protections are developed to strengthen the hardware for implementing these circuits. In this paper, we presented a design methodology that uses XADCs in the FPGA as a countermeasure to detect and thwart non-invasive active attacks, in particular those based on the supply voltage, temperature, and electromagnetic pulses. The proposed solution offers the possibility of using FPGA resources for protection with minimal resource cost in the case of FPGA technologies because the XADCs are implemented in the FPGA itself. Several temperature, voltage, and EM tests were performed, and the results show that once the ranges of temperature and voltage were defined, the scheme was able to detect any variation when the circuit was outside of its operating range. On the other hand, the results showed no frequency degradation because the scheme was implemented in parallel with the cryptocircuit, and in the case of the area overhead, the penalty was due to the attack response, which was 1%. In addition, this scheme is useful for different types of analysis because it is very versatile and can be implemented together with any cryptosystem. It should be noted that such schemes and methodologies are also applicable to Application-Specific Integrated Circuit (ASIC) technologies if an ADC is included in the circuit. This is an important point for future work, as the analog design of an ADC in ASICs will allow us to determine its impact on the design, as well as to study the variability of results with respect to those that can be obtained in FPGAs.

## Figures and Tables

**Table 1:** Classification of DFA attacks on AES.

Reference	Attack on State	Attack on S-Box	Attack on KeySchedule	Type of Attack	Attack in Round
[17]	Yes	No	Yes	S. bit|S. byte	8 and 9
[18]	Yes	∼	No	M. bit (same byte)	7, 8, and 9
[19]	Yes	Yes	No	S. byte	8
[20]	Yes	∼	No	S. byte— random	During encryption

S. bit and M. bit = single-bit and multi-bit, respectively. S. byte and M. byte = single-byte and multi-byte, respectively.

**Table 2:** Operating ranges for supply voltage.

$V_{\mathrm{CCINT}}$ (V)		$V_{\mathrm{CCBRAM}}$ (V)		$V_{\mathrm{CCAUX}}$ (V)	
Min.	Max.	Min.	Max.	Min.	Max.
0.88	1.05	0.88	1.05	0.88	1.8

**Table 3:** Comparison with different detection schemes.

Scheme	Type of Protection	Area Overhead	Frequency Degradation	Attack Detection	Technology
Unprotected	None	1	1		Artix-7
[28]	Information	1.08	0.7	75.6%	Virtex 1000
[29]		1.44	NIA	99.12%	NIA
[30]		1.73	0.64	88%	NIA
[31]		1.40	NIA	97%	NIA
[32]		1.32	0.97	97%	Virtex II
[33]		1.77	0.86	90%	Virtex E
[34]		1.25	0.88	98%	Virtex 5
[35]	Hardware	1.87	1	100%	Virtex 5
[36]	Temporal	1.07	0.83	100%	Virtex 4
[37]	Combination	1.38	0.78	100%	Virtex 6
[38]		1.58	0.83	93.75%	Spartan 3
Our Scheme	-	1.01	1	100%	Artix-7

NIA = No Information Available. Artix, Virtex and Spartan versions belong to Xilinx Inc. from San Jose, CA, USA.

## Data Availability

The original contributions presented in the study are included in the article, further inquiries can be directed to the corresponding author.

## Abbreviations

The following abbreviations are used in this manuscript:

ADC	Analog-to-digital converter
AES	Advanced Encryption Standard
AMS	Analog mixed signal
ASIC	Application-Specific Integrated Circuit
DES	Data Encryption Standard
DFA	Differential Fault Analysis
EM	Electromagnetic
EMFI	Electromagnetic Fault Injection
EMP	Electromagnetic pulse
FF	Flip-flop
FIA	Fault injection attack
FPGA	Field-Programmable Gate Array
IFA	Ineffective Fault Attack
IoT	Internet of Things
NIST	National Institute of Standards and Technology
PC	Personal Computer
RAM	Random-Access Memory
SCA	Side-Channel Analysis
XADC	Xilinx Analog-to-Digital Converter
